# A Survey on the Knowledge and Attitudes of Italian Medical Students toward Body Donation: Ethical and Scientific Considerations

**DOI:** 10.3390/jcm7070168

**Published:** 2018-07-09

**Authors:** Rosagemma Ciliberti, Matteo Gulino, Valentina Gazzaniga, Fabio Gallo, Valerio Gaetano Vellone, Francesco De Stefano, Pierluigi Santi, Ilaria Baldelli

**Affiliations:** 1 Section of Forensic Medicine and Bioethics, Department of Health Sciences, University of Genoa, Via De Toni 12, 16132 Genova, Italy; rosellaciliberti@yahoo.it (R.C.); fdestefano@unige.it (F.D.S.); 2 Department of Medico-Surgical Sciences and Biotechnologies, Sapienza University of Rome, Corso della Repubblica, 79, 04100 Latina, Italy; valentina.gazzaniga@uniroma1.it; 3 Section of Biostatistic, Department of Health Sciences, University of Genoa, Via Antonio Pastore 1, 16132 Genova, Italy; fabio.gallo@unige.it; 4 Pathology Accademy Unit, San Martino Hospital, 16132 Genova, Italy; valerio.vellone@unige.it; 5 Department of Surgical Sciences and Integrated Diagnostics (DISC), University of Genoa, 16132 Genova, Italy; plsanti@unige.it (P.S.); ilaria.baldelli@unige.it (I.B.); 6 Plastic and Reconstructive Surgery Unit, Ospedale Policlinico San Martino, 16132 Genova, Italy

**Keywords:** post mortem body donation, cadaver, ethics, students’ attitudes, anatomy education, medical education, cadaver lab, unclaimed bodies

## Abstract

Post mortem body donation (PMBD) for medical training and research plays a key role in medical-surgical education. The aim of this study is to evaluate Italian medical students’ awareness and attitudes regarding this practice. A questionnaire was sent to 1781 Italian medical students (MS). A total of 472 MS responded: 406 (92.91%) had a strongly positive attitude to PMBD, while 31 (7.09%) were not in favor. The majority of subjects were Catholic (56.36%), while 185 and 21 subjects, said that they did not hold any religious beliefs, or were of other religions, respectively. Multivariate analysis showed significant associations (*p*-values < 0.05) between PMBD and religion, as well as perceptions of PMBD as an act of altruism, a tool for learning surgical practices, body mutilation, and an act contrary to faith. Although Italian MS believed cadaver dissection to be an important part of their education, they did not know much about it and had not received training on this altruistic choice. As future doctors, MS can play an important role in raising public awareness of the importance of PMBD for medical education and research. Specific educational programs to improve knowledge of this topic among MS are needed.

## 1. Introduction

The scientific literature is in agreement on the importance of autopsy and cadaver dissection in medical student training and the professional upgrading of specialists [1,2]. Cadaver labs play an important educational role in the teaching not only of anatomy, but also of pathology and of surgical methodology [3]. Indeed, physicians, and dentists need to acquire theoretical knowledge and good technical skills before performing surgical procedures on living patients. However, the protection of patients and the ethical principle of non-maleficence require the acquisition of practical as well as theoretical skills. Dissection-based teaching is a focal point in the integration and correlation of basic and clinical medical knowledge. Through the dissection of corpses, students and physicians can have a direct view of the three-dimensionality of human tissues and direct contact with the structure of tissues, and can understand the differences between normal and pathological conditions [4].

In Italy, anatomical dissection has, however, become extremely rare, owing to the scant availability of corpses donated for study and research. Clinical autopsies are also becoming increasingly rare as they are often considered expensive, time-consuming, a potential source of medico-legal dispute, and a violation of the dignity of the deceased [5,6]. Consequently, Italian surgeons are often forced to import human cadavers or to go abroad to attend training courses, which involves high costs and professional and personal discomfort [7,8]. However, among anatomists, there seems to be considerable consensus that human bodies for educational purposes should be obtained exclusively from donors who during their lifetime legally stipulated an intention to donate their body to science [9,10].

Post mortem body donation (PMDB) is an important source of cadavers worldwide and provides a valuable opportunity to carry out research or educational activities in medicine and surgery. It is defined as an informed and free act of donating one’s whole body after death for medical education and research. This altruistic choice of high moral value could be favored by educating medical students (MS) with regard to PMBD. Indeed, as MS are the future health care providers for the community, they will be able to play a central role in raising public awareness of PMBD. In addition, they themselves constitute a potential donor population.

In other countries, research has already examined the attitudes and points of view of MS towards PMBD for study and research purposes [11,12,13]. Studies on medical and healthcare students’ attitudes toward body donation have indicated that donation is mainly motivated by altruism, while the main reasons for not donating include lack of information and religious factors [14]. In addition to this, studies have indicated that student attitudes to whole body donation are influenced by dissection [15]. Moreover, MS are deemed to be ideally suited to informing the public of the advantages of altruistic body donation [16,17]. To our knowledge, however, no studies have yet investigated attitudes towards this issue among Italian MS.

The purpose of the present pilot study was to ascertain the state of knowledge and perceptions of PMBD in a sample of Italian MS and to identify factors that can influence it. The results of this survey may be regarded as the starting point for further research and for the implementation of educational projects aimed at increasing the level of medical students’ awareness of this significant practice.

## 2. Experimental Section

The survey was conducted at the School of Medical and Pharmaceutical Sciences of the University of Genoa in December 2016, and involved both students enrolled in all years of the school and those who were behind schedule with their exams. All the respondents had completed an anatomy course before the survey was administered. Participants (*n* = 1781) were contacted via e-mail and received a link whereby they could access a completely anonymous self-administered questionnaire that could be filled in directly online. Participation was absolutely voluntary. The questionnaire, consisting of items concerning the student’s general data (age, sex, nationality, religion, academic course, and year attended) and 18 detailed closed questions (Q), was created by a panel of experts (a bioethicist, surgeon, pathologist, anatomy expert, lawyer, psychologist, and biostatistician), and gathered information on the knowledge of and attitudes towards PMBD, and the factors affecting these.

In this pilot study, age was categorized into three classes according to the tertile distribution (i.e.: 21 and 23 years as cut-off values). Each answer to Q1–Q18 was classified as for or against PMBD (encoded as “yes” or “no”). This was a single-center observational study, approved by the Regional Ethics Committee of Liguria, Genoa, Italy (P.R. 410REG2016).

### Statistical Analysis

Continuous variables are reported as means and standard deviations (SD), and categorical variables as the number and/or percentage of subjects. To identify and describe the underlying latent construct of the questionnaire, multiple correspondence analyses were carried out. Question Q4 was taken as the primary outcome measurement. In order to assess the association between PMBD and demographic and social factors, a univariate analysis was performed by means of logistic regression. Those covariates with a *p*-value < 0.05 were then selected for multivariate analysis, in which PMBD was the dependent variable. Multivariate analysis was also performed by means of logistic regression, and model selection was carried out by means of the Akaike information criterion. The likelihood ratio (LR) test was used to test statistical significance. Differences with a *p*-value less than 0.05 were deemed significant and data were acquired and analyzed using the R v3.3.2 software environment [18].

## 3. Results

The link to the questionnaire was sent to 1781 students (794 male and 987 female) enrolled at the School of Medical and Pharmaceutical Sciences of the University of Genoa.

A total of 472 (264 female) Italian MS completed the questionnaire and were evaluated in this study. The social and demographic characteristics of the study participants are summarized in Table 1. The mean age was 22 years (SD = 2.01; range, 19–42 years). Concerning religion, the majority of subjects were Catholic (56.36%), while 185 and 21 subjects said that they did not hold any religious beliefs or were of other religions, respectively. Of the 472 students, 284 (60.17%) were enrolled in the third year of university, while 63 (13.35%), 44 (9.32%), 39 (8.26%), and 37 (7.84%) students were registered in the sixth, second, fourth, and fifth year of university, respectively. Five students (1.06%) were behind in their university schedule.

**Figure 1:**
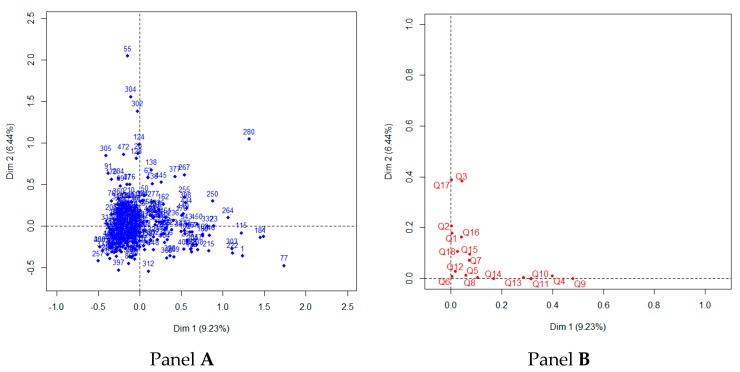
Multiple correspondence analysis. Observations and variables factor map are reported in panels (**A**) and (**B**), respectively.

Descriptive statistics of the clinical and demographic characteristics that displayed a significant association with PMBD are reported in Table 2. Specifically, 31 (7.09%) students out of 472 were against PMBD. The multiple correspondence analysis (Figure 1) showed no significant underlying latent constructs. On univariate analysis, religion had a significant effect on PMBD: Q5, Q7, Q8, Q9, Q10, Q11, Q13, and Q14 (*p*-values < 0.05).

The univariate variables with a *p*-value < 0.05 were then considered in the multivariate analysis (Table 3), which showed a statistically significant effect of religion on PMBD: Q7, Q8, Q9, and Q10 (*p*-values: 0.0021, 0.0145, 0.0255, 0.0021, and 0.0046, respectively). Specifically, students who did not hold any religious beliefs were about 5.9 (odds ratio, OR = 5.89) times more likely to be in favor of body donation than Catholic students. The probability of a pro-donation attitude among students who answered “yes” to questions Q7 and Q8 was about 3.9 and 3.8 times higher than among those who answered “no” (OR: 3.91 and 3.82, respectively). Finally, students who answered “yes” to questions Q9 and Q10 were about 80% and 84% less likely to have a pro-donation attitude than those who answered “no”, respectively (ORs: 0.20 and 0.16, respectively).

## 4. Discussion

The number of students who completed the questionnaire fell within the expected range, according to the data available in the literature for e-mail surveys [19]. The age range of the study population also appeared to be appropriate, covering the main period in which students attend university (Table 1).

As in other countries, the majority of MS agreed that autopsies and corpse dissection were useful in medical practice and education [4]. However, it emerged that a considerable percentage of students had scant knowledge of this issue. Similarly, a very high percentage of students also declared that they had not been introduced to the topic of organ and tissue donation during their academic training. (Table 1).

This cognitive gap is particularly critical in view of the sample considered (medical students) and the type of training that should be provided on this important topic throughout the curriculum.

Despite this major shortcoming, the high correlation between PMBD and its perception as “a morally significant act of altruism”, as shown by our data, confirms a generally positive attitude towards PMBD among the respondents (Table 2).

While a slight majority of students declared themselves to be Catholic, those who professed no religious faith were sufficiently represented. Analysis of the data also revealed that PMBD was not regarded as an act contrary to religious faith (Table 1); this is in agreement with the generally favorable stance of almost all religions in the world, which support and encourage donation [20,21]. It is noteworthy, however, that atheists and agnostics expressed a 6-fold greater approval of PMBD than those who professed to be Catholics (Table 3). This propensity shows that there are strong motivations, other than spiritual ones, that support this altruistic choice; these are probably linked to the awareness of the great ethical value of body donation and to a widespread sentiment of civic altruism and human solidarity.

As in other studies, Italian MS generally declared a favorable attitude towards organ donation [22]. The greater favorability towards organ donation than towards PMBD can probably be explained by the greater awareness and regulation of organ donation and related issues in Italy over recent decades (Law n. 91/1999, pertaining to provisions concerning the explanation and transplantation of human tissues and organs). With regard to PMBD, by contrast, there is a lack of specific legislation concerning the ownership of cadavers, the terms and conditions of donors’ informed consent, and the preservation of bodies [23]. As few bodies are donated voluntarily, the use of unclaimed cadavers may expose vulnerable individuals to dissection in absence of their consent [24]. Despite the lack of specific legislation regulating PMBD, many centers for the collection of body donations have been established and important programs for the donation of bodies have been initiated [25]. PMBD for scientific purposes continues to be very rare in Italy. One reason for this is probably that donating the body for the purpose of scientific study is perceived as being less useful than organ donation, which has “life-saving” connotations.

Moreover, unlike organ donation, body donation may elicit irrational concerns and fears [26]. The idea that one’s body can be dissected may create psychological obstacles, which can only be overcome by emphasizing the importance of donation and its scientific and medical utility.

The promotion of a pro-PMBD culture and the adoption of measures to regulate this practice for scientific purposes may not only improve physicians’ anatomical and surgical education, but also significantly reduce the number of animals sacrificed [27,28]. Such policies may consequently narrow the gap between Italy and many other countries where there is a good availability of donated bodies for educational purposes. As physicians can play a pivotal role in promoting PMBD and also be a good vehicle of information for patients and relatives, students should be directly trained in this matter.

Although the vast majority of respondents did not consider PMBD as an insult to the human body, we must underline that those who did were about 80% less likely to be in favor of PMBD. Kharkar and Dase pointed out that students’ concerns that their bodies might not be handled correctly prevent body donation. The ceremony with which some research institutes express their gratitude for the gift of body donors plays an important role and raises students’ awareness of the need to treat donors’ bodies respectfully [29,30,31].

Fear that death has not been adequately verified or that there is a chance of awakening after death has been declared can affect the willingness to donate the body. With regard to brain death, the educational aspect is of fundamental importance to the role that the physician assumes within the community.

An important issue related to PMBD concerns the relationship with the family. Indeed, our data indicate that a high percentage of students associate PMBD with discomfort for their families.

In conclusion, MS perceive PMBD as an altruistic and useful act. The results of the study highlight the importance of a culturally sensitive approach to students in their curricula. They also highlight the need for well-organized and informative body donation programs [14]. However, several barriers prevent the principles of social solidarity contained in the Italian legal system from being put into effect. In particular, the failure to provide MS with adequate education on PMBD suggests a lack of a scientific and academic strategy for making PMBD a common and shared practice. We believe that orientating the public towards this practice is of high moral and medical value. Media and other social bodies could take an important role in promoting this generous act in our country.

## 5. Study Limits

The surveyed student population had not attended any compulsory academic course involving anatomical dissection, and the few optional courses that involve autopsies are loaded with requests that fail to be met. The views expressed are therefore abstract and purely theoretical. The possibility of attending a human body dissection course could affect students’ opinions. In most studies, however, the desire to donate one’s body to science has been seen to decrease after attendance of an anatomical dissection course for a few weeks [32]. This trend, however, disappears during post-graduate training, owing to the increasing awareness of the professional and social utility of PMBD [33]. Another important limitation of the study is the response rate to the questionnaire (26.5%); this situation may have introduced a positive bias whereby students who were most sensitive to the issue responded to the questionnaire. However, this rate is in line with the data available in the literature on e-mail surveys [19]. Finally, the questionnaire used in this pilot study will require further studies in order to allow its validation.

## Figures and Tables

**Table 1:** Social and demographic characteristics of study participants. The results are expressed as means with standard deviations or as numbers of subjects with percentages.

Characteristic	Overall
**Age**	
≤21	189 (40.04%)
22–23	167 (35.38%)
≥24	116 (24.58%)
**Academic year**	
2	44 (9.32%)
3	284 (60.17%)
4	39 (8.26%)
5	37 (7.84%)
6	63 (13.35%)
Behind the study schedule	5 (1.06%)
**Gender**	
*Female*	264 (55.93%)
*Male*	208 (44.07%)
**Religion**	
Catholic	266 (56.36%)
None	185 (39.19%)
Other	19 (4.03%)
Muslim	1 (0.21%)
Orthodox	1 (0.21%)
**Specialization**	
Medical	317 (67.16%)
Surgical	128 (27.12%)
Diagnostic	27 (5.72%)
Do not know	102 (21.66%)

**Table 2:** Significant results on univariate analysis.

	Human Body Donation		Univariate Analysis	
Characteristic	No 31 (7.09%)	Yes 406 (92.91%)	ODDS RATIO (OR) (95% CI)	*p*-Value
**Religion**				0.0160
Catholic	23 (8.65%)	243 (91.35%)	1	
None	6 (3.24%)	179 (96.76%)	2.75 (1.18:7.31)	
Other	3 (14.29%)	18 (85.71%)	0.46 (0.14:1.85)	
**Q5:** Do you support organ donation?				0.0004
No	3 (75%)	1 (25%)	1	
Yes	29 (6.2%)	439 (93.8%)	33.12 (5.24:350.2)	
**Q7:** Do you regard post-mortem body donation as a morally significant act of altruism?				0.0012
No	9 (18%)	41 (82%)	1	
Yes	23 (5.45%)	399 (94.55%)	4.49 (1.87:10.16)	
**Q8:** Do you regard cadavers as a fundamental source for learning basic and advanced surgical practices?				0.0006
No	11 (22%)	39 (78%)	1	
Yes	21 (4.98%)	401 (95.02%)	5.03 (2.08:11.51)	
**Q9:** Do you associate post-mortem body donation with violation of the body?				<0.0001
No	17 (4.04%)	404 (95.96%)	1	
Yes	15 (29.41%)	36 (70.59%)	0.1 (0.05:0.22)	
**Q10:** Do you regard post-mortem body donation as an act contrary to your religious faith?				<0.0001
No	24 (5.32%)	427 (94.68%)	1	
Yes	8 (38.1%)	13 (61.9%)	0.09 (0.03:0.24)	
**Q11:** Do you associate post-mortem donation with fear?				0.0001
No	16 (4.19%)	366 (95.81%)	1	
Yes	16 (17.78%)	74 (82.22%)	0.21 (0.1:0.44)	
**Q13:** Do you associate your choice regarding post-mortem body donation as a disadvantage for your family?				0.0006
No	8 (2.88%)	270 (97.12%)	1	
Yes	24 (12.37%)	170 (87.63%)	0.26 (0.11:0.56)	
**Q14:** Do you think that anatomical dissection of corpses in your course would be important?				0.0003
No	8 (32%)	17 (68%)	1	
Yes	24 (5.37%)	423 (94.63%)	7.24 (2.66:18.4)	

Descriptive statistics and a summary of significant results on univariate analysis are reported. Characteristic: variable taken into account; OR (95% CI): odds ratios with 95% confidence interval; *p*-value: likelihood ratio (LR) *p*-value.

**Table 3:** Multivariate analysis (*n* = 349). Characteristic: variable taken into account; OR (95% CI): odds ratios with 95% confidence interval; *p*-value: LR *p*-value.

Characteristic	OR (95% CI)	*p*-Value
**Religion**		0.0021
Catholic	1	
None	5.89 (1.61:33.84)	
Other	0.26 (0.07:1.24)	
**Q7**		0.0145
No	1	
Yes	3.91 (1.33:11)	
**Q8**		0.0255
No	1	
Yes	3.82 (1.19:11.48)	
**Q9**		0.0021
No	1	
Yes	0.20 (0.08:0.55)	
**Q10**		0.0046
No	1	
Yes	0.16 (0.05:0.55)	
